# Mapping-by-sequencing using NGS-based 3′-MACE-Seq reveals a new mutant allele of the essential nodulation gene *Sym33* (*IPD3*) in pea (*Pisum sativum* L.)

**DOI:** 10.7717/peerj.6662

**Published:** 2019-04-02

**Authors:** Aleksandr I. Zhernakov, Oksana Y. Shtark, Olga A. Kulaeva, Jaroslava V. Fedorina, Alexey M. Afonin, Anna B. Kitaeva, Viktor E. Tsyganov, Fabian Afonso-Grunz, Klaus Hoffmeier, Björn Rotter, Peter Winter, Igor A. Tikhonovich, Vladimir A. Zhukov

**Affiliations:** 1 All-Russia Research Institute for Agricultural Microbiology, St.Petersburg, Russia; 2 GenXPro GmbH, Frankfurt am Main, Germany; 3 St.Petersburg State University, St.Petersburg, Russia

**Keywords:** Mapping-by-sequencing, Next generation sequencing, RNA-Seq, Massive analysis of cDNA Ends, Pea, Symbiotic genes

## Abstract

Large collections of pea symbiotic mutants were accumulated in the 1990s, but the causal genes for a large portion of the mutations are still not identified due to the complexity of the task. We applied a Mapping-by-Sequencing approach including Bulk Segregant Analysis and Massive Analysis of cDNA Ends (MACE-Seq) sequencing technology for genetic mapping the *Sym11* gene of pea which controls the formation of symbioses with both nodule bacteria and arbuscular-mycorrhizal fungi. For mapping we developed an *F*_2_-population from the cross between pea line N24 carrying the mutant allele of *sym11* and the wild type NGB1238 (=JI0073) line. Sequencing libraries were prepared from bulks of 20 plants with mutant and 12 with wild-type phenotype. MACE-Seq differential gene expression analysis between mutant-phenotype and wild-type-phenotype bulks revealed 2,235 genes, of which 514 (23%) were up-regulated and 1,721 (77%) were down-regulated in plant roots inoculated with rhizobia as a consequence of *sym11* mutation. MACE-Seq also detected single nucleotide variants between bulks in 217 pea genes. Using a novel mathematical model we calculated the recombination frequency (RF) between the *Sym11* gene and these 217 polymorphic genes. Six genes with the lowest RF were converted into CAPS or dCAPS markers and genetically mapped on the complete mapping population of 108 *F*_2_-plants which confirmed their tight linkage to *Sym11* and to each other. The *Medicago truncatula* Gaertn. (Mt) homologs of these genes are located in a distinct region of Mt chromosome 5, which corresponds to linkage group I of pea. Among 94 candidate genes from this region only one was down-regulated—the pea *Sym33* homolog of the Mt *IPD3* gene which is essential for nodulation. Sequencing of the *Sym33* allele of the N24 (*sym11*) mutant revealed a single nucleotide deletion (c.C319del) in its third exon resulting in a codon shift in the open reading frame and premature translation termination. Thus, we identified a novel mutant allele *sym33-4* most probably responsible for the mutant phenotype of the N24 (*sym11*) line, thereby demonstrating that mapping by MACE-Seq can be successfully used for genetic mapping of mutations and identification of candidate genes in pea.

## Introduction

Next generation sequencing (NGS) currently revolutionizes forward genetics since massive sequencing offers a wide range of analysis schemes depending on the genetic background of the species, the type of genetic alteration, the phenotype, etc. In particular, it may be implemented for the direct search of DNA variants in genomes from individuals possessing the features of interest or for genetic mapping of genes responsible for these features—a technique named mapping-by-sequencing (MBS).

Mapping-by-sequencing is most conveniently combined with “bulked segregant analysis” (BSA) developed for identifying genetic markers linked to a trait-governing gene (Giovannoni et al., 1991; Michelmore, Paran & Kesseli, 1991). It involves bulking of a subset of individuals from the mapping population that share a target feature. BSA then assumes that the chromosomal region where the trait-conferring gene resides is the only region for which the bulks are different and that, thus, markers that differentiate between the bulks are located in this region and are therefore genetically linked with the target gene(s).

Next generation sequencing was first combined with BSA for identification of an *Arabidopsis thaliana* mutant allele. The experiment included 22-fold genome coverage sequencing of one library prepared from the pool of 500 mutant *F*_2_-plants obtained after crossing the mutant line with a polymorphic line (Galvão et al., 2012). This approach was highly effective and detected the non-synonymous mutation within the region by mapping the NGS reads to the reference genome sequence. Subsequently, a nucleotide variation was confirmed as the causal mutation for the phenotype.

Methods used for genome complexity reduction such as exome sequencing, RAD-sequencing (Restriction-site associated DNA) and RNA sequencing (RNA-Seq) are also applicable for MBS. Exome sequencing still requires the genome information and costly DNA-capturing arrays (Mascher et al., 2013), while RNA-Seq can be applied to all species for identification of linked markers (Trick et al., 2012) and mutation discovery, even though it allows to detect only mutation(s) in transcribed sequences. RNA-Seq further requires high read coverage to reliably detect polymorphisms in lowly expressed sequences. Massive Analysis of cDNA Ends (MACE-Seq) is a modification of RNA-Seq which generates only a single sequence from the 3′-end of each poly-adenylated transcript (Zawada et al., 2014; Zhernakov et al., 2017). As sequencing reads are concentrated at a small part of the transcript, a relatively small number of sequences is sufficient for reliably covering polymorphisms in most cases. MACE-Seq has already been successfully combined with BSA to genetically map the stem rust resistance locus in perennial ryegrass (Bojahr et al., 2016), and anthracnose resistance in lupin (Fischer et al., 2015). Recently, we have shown that MACE-Seq is a convenient approach for polymorphism discovery and genetic marker design also in pea (*Pisum sativum* L.)—a species of high importance for both applied and fundamental studies, which genome is not sequenced yet (Zhernakov et al., 2017). Therefore, we decided to test if MACE-Seq is appropriate for mapping pea genes with unknown localization in genome.

Here, we present the results of mapping of the pea gene *Sym11* of the mutant line N24 followed by candidate gene analysis using the combination of MBS approach including bulk segregation analysis and MACE-Seq technology (MB-MACE). N24 was isolated as a non-nodulating (Nod^−^) mutant (Kneen, Weeden & LaRue, 1994), but the nodulation phenotype of N24 is not yet studied in details. The mycorrhizal phenotype of N24 suggests that the mutant is impaired in penetration of the rhizodermis by mycorrhizal fungi; indeed, N24 exhibits rare/occasional events of internal fungal colonization accompanied by an abnormally intense external mycelium development (Shtark et al., 2016). The only mapping of the *sym11* gene using morphological and isozyme markers was performed almost 25 years ago (Kneen, Weeden & LaRue, 1994). Here, we now show that *Sym11* is located in pea linkage group I, and most likely is a novel allele of the symbiotic gene *IPD3* (known as *Sym33* in pea) (Ovchinnikova et al., 2011), since the N24 line encodes a truncated protein as a consequence of a single-nucleotide deletion in the third exon of the *Sym33* (*IPD3*) gene sequence.

## Materials and Methods

The overall scheme of the work is outlined in Fig. S1.

### Mapping populations

The non-nodulating pea mutant line N24 (JI3047, named in accordance to John Innes Centre SeedStor database, https://www.seedstor.ac.uk/) carrying a mutation in the *sym11* gene was obtained from cultivar Sparkle (JI0427) by fast neutrons mutagenesis (Kneen, Weeden & LaRue, 1994). N24 (*sym11*) and its parental genotype Sparkle were kindly provided by Prof. T. A. LaRue (Boyce Thompson Institute for Plant Research Department of Plant Biology) in the 1990s and were further propagated in All-Russia Research Institute for Agricultural Microbiology (St. Petersburg, Pushkin, Russia). Since then both lines were selfed several times to multiply seeds and maintain their vitality. The mutant non-nodulating phenotype remained stable during a series of physiological experiments.

The N24 line was crossed in parallel with the wild-type pea lines NGB1238 (JI0073, also known as WBH1238, WL1238) and SGE (JI3023) (Kosterin & Rozov, 1993). *F*_1_ hybrid heterozygosity was confirmed by morphological markers. Generation *F*_1_(NGB1238 × N24) yielded a total of 108 *F*_2_-seeds, *F*_1_(SGE × N24) yielded 27 seeds. Screening of *F*_2_-populations was conducted as follows. Seeds were surface sterilized by concentrated sulfuric acid, rinsed 5–7 times in sterile distilled water and five seeds per a pot planted into two-litre pots. These were filled with sand supplied with nitrogen-free mineral nutrient solution (Borisov et al., 1997). At planting, the seeds were inoculated with one ml of a 10^6^ CFU suspension of the *Rhizobium leguminosarum* bv. *viciae* RCAM1026 strain (Afonin et al., 2017) per seed. Plants were watered as needed with autoclaved tap water and harvested 3 weeks after planting. After removing them from the pot, the root system of each plant was examined for nodulation and frozen in liquid nitrogen as soon as possible for further RNA extraction; several leaves per plant were individually collected from the shoots for further DNA extraction. The root samples were kept at −80 °C, the shoot samples at −20 °C till needed.

### Pooling, library preparation and sequencing of *F*_2_(NGB1238 × N24) root samples

Frozen roots were pooled into bulks using visually equal amounts of tissue from Fix^−^ (forming white nodules) or Nod^−^ (non-nodulating) phenotypes (four pools of *n* = 5, M-pools) as well as 12 plants from Nod^+^ (normal nodulating) phenotype (two pools of *n* = 6, W-pools). Pooled tissues were ground in liquid nitrogen, stabilized in RNAlater solution (Thermo Fisher Scientific, Waltham, MA, USA) and shipped to GenXPro GmbH, Frankfurt am Main, Germany, where RNA isolation, NGS-library preparation and sequencing was performed. Total RNA was isolated using the Nucleospin miRNA Kit (Macherey-Nagel GmbH & Co. KG, Düren, Germany) according to the protocol for isolation of total RNA from plant tissue. Library preparation was carried out with the MACE-Seq Kit (GenXPro GmbH, Frankfurt, Germany) and the obtained libraries were sequenced on an Illumina NextSeq 500 platform with single-end sequencing of 75 base pair reads. The relevant raw data is available in the short read archive from NCBI (PRJNA506113).

### Polymorphisms discovery

For polymorphisms discovery, we used the pea nodule transcriptome assembly (PNTA) (Zhukov et al., 2015) deposited at NCBI Transcriptome shotgun assembly database under accession GDTM. Trimmed and cleaned sequencing reads of each bulked library were mapped to the PNTA with the Bowtie2 software (version 2.1.0) (Langmead & Salzberg, 2012), SM-tag designating the bulk was added. Produced SAM-files were converted to BAM format and merged into a single file with the Samtools utility (version 0.1.18) (Li, 2011). Single nucleotide variant (SNV)-calling was performed with the FreeBayes tool (version 1.2.0) (Garrison & Marth, 2012) in the MACE-Seq sequences from Sparkle, NGB1238 and the respective M(utant) and W(ild type) bulks (Zhernakov et al., 2017). Nucleotide variants detected in the samples were excluded from the analysis if: (1) the QUAL parameter value was less than 20; (2) the variant was not an SNV (i.e., indel) or was a novel variant not seen in the parents; (3) the coverage depth in any library was less than 40.

### Linkage estimation

To evaluate the linkage of the detected SNVs with the causal gene *sym11*, we preferred to not only statistically compare the observing SNVs frequency in both groups (normal phenotype and mutant phenotype) using *G*-test but to estimate the recombination frequency (RF) (α) between the loci.

Recombination frequency directly defines how many alleles of one of the parents we can expect in an offspring sample. Consider a discrete random variable *G* that represents the number of L-alleles (opposite to l-allele) in a bulked sample consisting of *n* *F*_2_-individuals. The probability mass function of *G* is

$$P\left( {G = g} \right) = \mathop \sum \limits_{2k + h = g} {{n!} \over {k! \cdot h! \cdot \left( {n - k - h} \right)!}} \cdot {p_{{\rm{LL}}}}^k \cdot {p_{{\rm{Ll}}}}^h \cdot {p_{{\rm{ll}}}}^{n - k - h}$$

Where *k*—number of LL-individuals, *h*—number of Ll individuals, ${p_{{\rm{LL}}}} + {p_{{\rm{Ll}}}} + {p_{{\rm{ll}}}} = 1$ are probabilities to select LL- Ll- or ll-individual from the general population. For a pool consisting of individuals that are homozygous at locus T (coming from the same parent as L), the probabilities *p*_LL_, *p*_Ll_ and *p*_ll_ took values (1−**α**)^2^, 2 α*(1−**α**) and **α**^2^, respectively, where **α** is the RF between loci T and L.

As we work with bulked samples we cannot know the exact value of *G* in a pool, so we made several assumptions for connecting the RF (**α**) with the observed number of mutant-line-originated reads (*M*) among all ***c*** reads covering a SNV. First, we assume that the *M* is binomially distributed with parameters *c*—coverage and Γ —percent of mutant-line-originated fragments in a sequencing library.

$$M \sim {\rm{Bin}}\left( {c,\Gamma } \right)$$

Γ directly depends on the number of mutant alleles in the pool: $\Gamma \simeq {G \over {2 \cdot n}}$. The main source of variance for Γ is unevenness of mixing. For large samples (*n* > 100) we can neglect this variance, but for small ones it may significantly contribute to the variance of the estimated value of RF. To model this variance we assume that Γ is distributed according to a Beta-distribution with parameters reflecting the ratio of alleles of both parents in a bulk considering mixing accuracy.

$$\Gamma \sim {\rm{Beta}}\left( {G \cdot {\rm{\xi }};\left( {2 \cdot n - G} \right) \cdot {\rm{\xi }}} \right)$$

where ξ —is the mixing accuracy coefficient.

Based on these assumptions we construct the maximal likelihood function maximizing the probability of getting the observed number of reads in all samples at unknown RF **α** for an SNV.

$$L = \mathop \prod \limits_{{\rm{pools}}}^ P({M_i} = m|{\rm{\alpha }})$$

Using this ML-function we also calculated 80% confidence interval for each estimation of α.

Also, based on the proposed model, SNVs for which one or more libraries displayed an improbable ratio of inherited alleles were excluded from the linkage analysis.

### Co-segregation analysis

For verification of genetic linkage and estimation of RF values between the *sym11* gene and other genetic loci, we conducted a co-segregation analysis on both the mapping populations *F*_2_(NGB1238 × N24) and *F*_2_(SGE × N24). Genomic DNA was individually extracted from frozen leaves of *F*_2_-plants using CTAB buffer. Based on the identified SNVs between an outcross line Sparkle (N24) and NGB1238 in pea transcripts (see Table 1) we developed CAPS and dCAPS markers applicable for genotyping (see Table 2). PCR primers were designed using the sequences of the transcripts from PNTA and similar pea transcriptome sequences publically available in the Nucleotide Collection of the NCBI database (https://www.ncbi.nlm.nih.gov/nuccore/), with the help of the online tool Primer-BLAST (Ye et al., 2012). The exon-intron structures of the pea transcripts were modeled by aligning their sequences with the sequences of orthologous genes of *Medicago truncatula* Gaertn. (version Mt4.0, http://www.phytozome.org) and were taken into account for primer design. The restriction endonucleases were selected by screening the polymorphic sequences by an in-house script. Fragments were amplified using the ready-to-use solution for PCR ScreenMix-HS (Evrogen, Moscow, Russia) and then digested by the selected restriction endonuclease: MboII, FastDigest Tru1I (Thermo Fisher Scientific, Waltham, MA, USA), BtsCI, Hpy166II (New England Biolabs, Ipswich, MA, USA), BssECI (SibEnzyme, Novosibirsk, Russia). Digestion patterns were analyzed with use of the microchip electrophoresis system MultiNA (Shimadzu, Kyoto, Japan).

**Table 1:** Pea genes that were designated as the closest to the *Sym11* gene by MB-MACE analysis, and the estimated recombination frequency between each gene and *Sym11*.

Name	Contig Id	Presumptive ortholog in *M. truncatula*	Desc1ription	Recombination frequency estimate with confidence interval
ZFP	GDTM01006086	Medtr5g026530	Zinc finger protein, putative	0.0256 (0–0.0727]
Upl	GDTM01054653	Medtr5g045520	Ureide permease-like protein	0.0669 (0–0.1259]
RibL13l	GDTM01018587	Medtr5g026750	60S ribosomal L13-like protein	0.0763 (0–0.1324]
AUXfp	GDTM01044379	Medtr5g030710	Auxin-responsive AUX/IAA family protein	0.0893 (0–0.1507]
PrOx	GDTM01024960	Medtr5g021060	Peroxidase family protein	0.1558 [0.0588–0.233]
sdhFP	GDTM01024974	Medtr5g020050	Succinate dehydrogenase (ubiquinone) flavoprotein subunit	0.1606 [0.0669–0.2369]

**Table 2:** CAPS-markers for pea genes presumptively linked with the *sym11* gene.

Marker Id	Primers	Endonuclease restriction	Applicability	
			N24 (Sparkle) vs. NGB1238	N24 (Sparkle) vs. SGE
ZFP	CTCCTTCCAAGCCAGATTGCGCAGTTTCTTATAGTTGTTTTAGG	MboIIGAAGA	Yes	No
Upl	TCGCTCAAAYTTACACTAAAGAAGTGGCCGTTGTTGTTCTCATGG	Tru1ITTAA	Yes	No
RibL13l	AGCCAATGTGCAGAGGTTGATGTCTTCCTTCTCTGCCTCG	BssECICCNNGG	Yes	Yes
AUXfp	GCAGGAATTAGAGGGTGCCTGAAGAGGTGGAGTCCGAACA	BtsCIGGATG	Yes	Yes
PrOx	TCAAACACACGCAACAATGCTGGGGTGAAATCAGGAAGCAC	Tru1ITTAA	Yes	No
sdhFP	CCCTCCTAAAGCTCGTGTGTAGATGCACAACTTAACATACAGTG	Hpy166IIGTNNAC	Yes	No
IPD3:C319del	GTTAATGTGTCTAATCAGCAACGAAACATCATTTGTCAAAAAGC	Tru1ITTAA	Yes	Yes

Recombination rates between the gene-based markers and *sym11* were calculated with the F2breed program (Zhernakov et al., 2017). Also, using the F2breed all genes were arranged into a linear genetic map.

For sequencing the candidate gene *Sym33* the genome region including 99% of the pea *IPD3* (GenBank: EF569222.1) open reading frame was PCR-amplified with Encyclo polymerase (Evrogen, Moscow, Russia). The target sequences for primer annealing used for amplification and sequencing are framed in Fig. S2.

### Differential gene expression

In order to determine the differential gene expression (DGE), the reads were trimmed using bbduk.sh tools from the bbmap package (Bushnell, 2014). After quality trimming the reads were mapped to the reference PNTA. Mapping was performed using bbmap suite with the following options: minid = 0.5 local, in order to compensate for the incompleteness of the assembly. The resulting mapping efficiency varied from 85% to 95%. Next, the read counts were analyzed using the DESeq2 package (Love, Huber & Anders, 2014). Samples were grouped according to phenotype (wild-types vs. mutants) and the DGE was calculated.

### Microscopy

Nodules from plants of population *F*_2_(NGB1238 × N24) were harvested 3 weeks after inoculation. They were fixed in fixative solution (4% paraformaldehyde, 0.1% Tween-20, 0.1% Triton X-100) in 1/4 MTSB (50 mM PIPES, five mM MgSO_4_·7H_2_O, five mM EGTA, pH 6.9). For optimal fixative penetration air from tissues was pumped out during 15 min at −0.9 bar using a VacuuBrand ME 1C vacuum pump (Vacuubrand, Wertheim, Germany) four times. Nodules were washed in MTSB 15 min four times, and molded in 3% agarose gel blocks. Longitudinal nodule sections (50 μm) were prepared using a microtome with a vibrating blade HM650V (Microm, Walldorf, Germany). Then sections were stained with 0.1 % Alcian blue (*w/v*). Sections were washed in TBS (50 mM TrisHCl, 150 mM NaCl, pH 7.5) three times for 5 min and mounted in Glycerol/TBS (1:1). The sections were examined on a microscope Axio Imager.Z1 (Carl Zeiss, Oberkochen, Germany) and photos were taken using a digital video camera Axiocam 506 (Carl Zeiss, Oberkochen, Germany).

## Results

**Figure 1:**
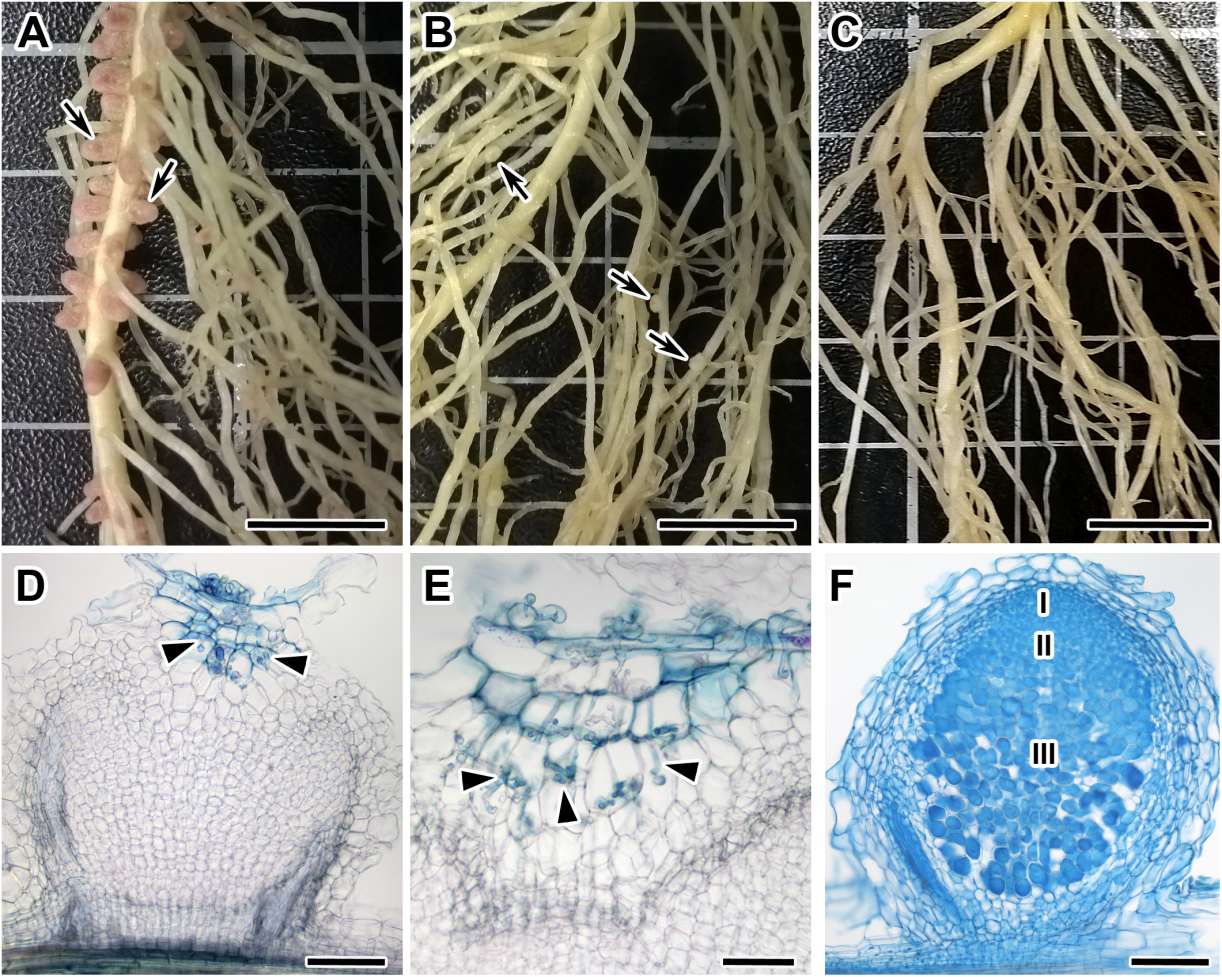
Root systems of segregants with Nod ^+^Fix^+^ and Nod ^+^Fix^−^ phenotypes from *F*_2_(NGB1238 × N24) and mutant N24 (*sym11*) and histological organization of formed nodules. Three weeks after inoculation with *Rhizobium leguminosarum* bv. *viciae* strain RCAM 1026. (A) Roots of a segregant with pink effective nodules (Nod^+^Fix^+^ phenotype) from *F*_2_(NGB1238 × N24). (B) Roots of a segregant with white ineffective nodules (Nod^+^Fix^−^ phenotype) from *F*_2_(NGB1238 × N24) (C) roots of mutant N24 (*sym11*) without any nodules (Nod^−^ phenotype). (D and E) Histological organization of white nodule of segregant with white ineffective nodules (Nod^+^Fix^−^ phenotype) from *F*_2_(NGB1238 × N24). (D) Longitudinal section of the nodule. Infection thread penetrated only the outer root cortex cells and the central part of the nodule remained empty. (E) Detailed view of ramified infection thread in the outer root cortex cells. (F) Histological organization of pink nodule of cv. Sparkle. Zones of nodules are designated by Roman numerals: I—meristem, II—infection zone, III—nitrogen fixation zone. Arrows indicate nodules, arrowheads indicate infection threads. Sections were stained with alcian blue. Scale bar (A–C) = 10 mm, (D, F) = 200 μm, (E) = 100 μm. Photo Credits: Shtark O. (photographies of root systems), Kitaeva A. (photomicrographies of nodules).

Among 108 *F*_2_-plants of the *F*_2_(NGB1238 × N24) mapping population, 77 plants formed normal pink nodules (Fix^+^) (Fig. 1A), 28 plants had flat white nodules (Fix^−^) (Fig. 1B), three plants were short and weak and did not form any nodules (Nod^−^). This Fix^−^ phenotype was surprising for us since earlier the N24 mutant line was described as Nod^−^ (Kneen, Weeden & LaRue, 1994; Borisov et al., 2004). In the Fix^−^ nodules of *F*_2_-plants infection threads penetrated only the outer root cortex cells and the central part of nodule remained empty (Fig. 1D). Control plants of both parental lines (siblings of plants used for crossing) as well as cv. Sparkle were grown at the same time and under the same conditions. The control plants of the NGB1238 line and cv. Sparkle formed pink nodules (Fix^+^) with typical histological organisation (Fig. 1E) and plants of the N24 line formed no nodules (Nod^−^). The formation of white nodules rather than no nodules in most of the *sym11*/*sym11* plants from *F*_2_-generation may be influenced by the genetic background eventually resulting in varying levels of plant hormones in roots of the parental lines used for the cross. The 3:1 segregation of the Fix^+^ phenotype (expected 81:27; observed 77:31; χ^2^-value = 0.79; *p*-value = 0.37) reflects the dominant character of the *Sym11* allele over the mutant *sym11*. Fix^+^ plants from *F*_2_-generation thus carried at least one dominant WT allele of *Sym11 (Sym11/Sym11 or Sym11/sym11)*, whereas Fix^−^ and Nod^−^ carried both recessive mutant alleles of *sym11*.

Roots of 19 Fix^−^ phenotype plants and one Nod^−^ phenotype plant (20 in total) were randomly divided into four pools (five plant samples per pool) and labeled M-pools (mutant). The same way roots of 12 randomly selected plants with clear Fix^+^ phenotype were mixed into two pools labeled as W-pools (wild-type). The MACE sequencing libraries were prepared from the root mixes and sequenced. The final number of obtained cleaned and filtered sequencing reads for each library varied from approximately 4.2–9.1 million of reads.

Previously analysis of polymorphisms between pea line Sparkle and NGB1238 by MACE sequencing revealed 8,848 potentially polymorphic sites in 3,436 transcripts (Zhernakov et al., 2017). After mapping the sequencing reads to the PNTA and following SNV-calling and filtering 778 polymorphic sites in 468 transcripts had been left for consideration. Of those, 445 SNV had been eliminated due to unlikely distributed ratios of reads originating from both parents. Finally, the linkage with the *sym11* gene had been estimated for 332 SNVs belonging to 217 contigs/genes (see Tables S1 and S2). For all the analyzed genes we calculated the point and interval estimates of the RF between these genes and the *sym11* gene and the LOD-score for the point estimate. For all the revealed genes we tried to find the orthologous genes in *M. truncatula* using BLASTN against the Mt4.0 CDS database (Tang et al., 2014).

**Figure 2:**
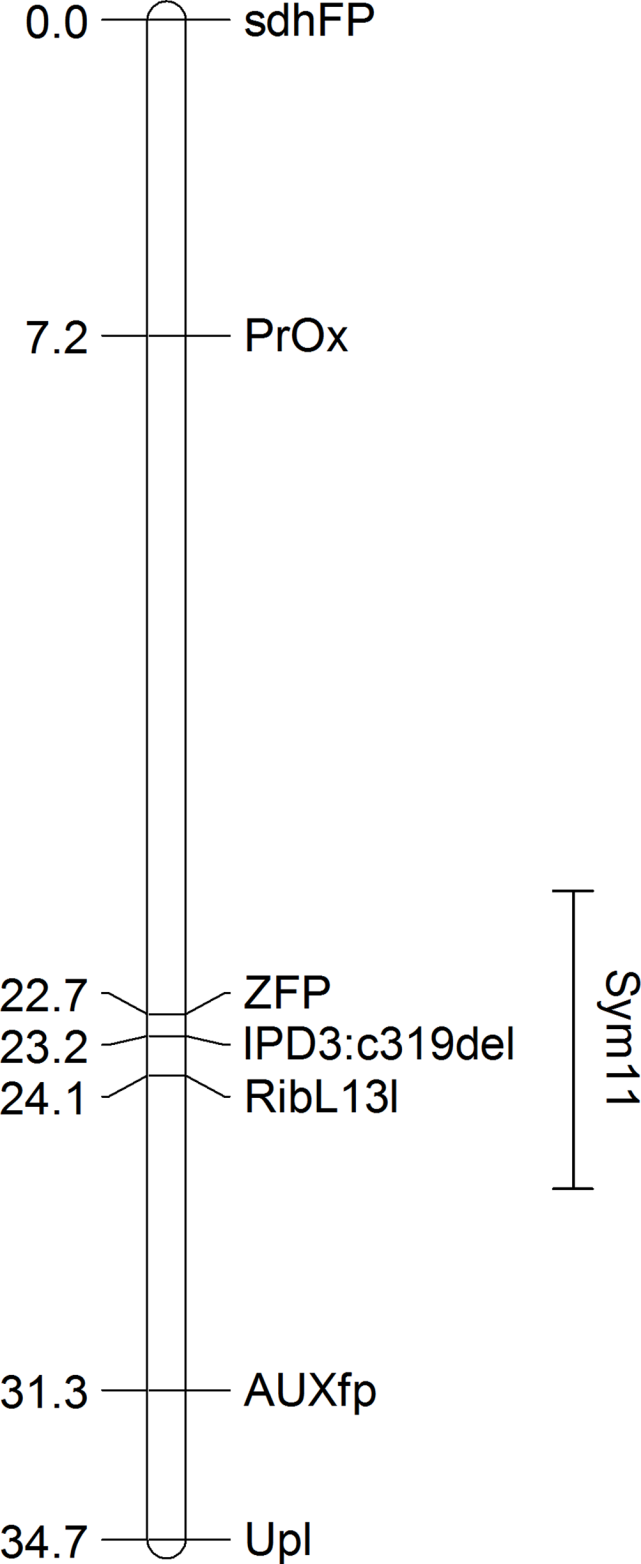
The genetic map constructed based on segregation of gene-based markers linked to *Sym11*. A bar represents the interval of the most probable position of *Sym11*, that was calculated on the base of genetic mapping using the *F*_2_(NGB1238 × N24) population.

Remarkably, when sorted by the point RF estimate (see Table 1), all of the presumed orthologs of the pea genes exhibiting the strongest linkage to the *Sym11* gene were located in a small region of chromosome 5 of *M. truncatula* (approx. 12.3 Mbp) that corresponds to LG I of pea according to the Pea Marker Database (Kulaeva et al., 2017). To confirm the localization of the *Sym11* gene in the outlined region of LG I, we designed either CAPS or dCAPS markers for the six pea genes which were defined as the closest to the *Sym11* gene (see Table 2). Markers were named according to the gene names of their respective Mt homologs. Segregation of the markers in the *F*_2_(NGB1238 × N24) mapping population proved their linkage with the *Sym11* gene (see Table 3). Fine mapping of the *Sym11* gene using the *F*_2_(NGB1238 × N24) mapping population of 108 plants showed that two markers ZFP (Zinc Finger protein, putative) and RibL13l (60S ribosomal L13-like protein) have the strongest genetic linkage with the *Sym11* gene. Both markers are approximately one recombination event per 31 plants or one cM away from the *Sym11* gene and three recombination events per 108 plants or 1.4 cM apart from each other. Unfortunately, this combination of markers could not unambiguously arrange the *Sym11* gene on a genetic map (see Fig. 2). The localization of the *Sym11* gene in this region was also confirmed with another mapping *F*_2_(SGE × N24) population of 27 plants (expected segregation 20.25:6.75; observed segregation 18:9; χ^2^-value = 1.0; *p*-value = 0.32), by analyzing the segregation of the two markers from this region: AUXfp (auxin-responsive AUX/IAA family protein) and RibL13l (see Table 3).

**Table 3:** Segregation of the *Sym11* gene and markers in the *F*_2_ outcross mapping populations.

Marker Id	Number of offsprings						Recombination frequency estimate	LOD	χ^2^—value
	Nod^+^ (normal *Sym11* phenotype, dominant)			Fix^−^/Nod^−^ (mutant *sym11* phenotype, recessive)					
	AA	Aa	aa	AA	Aa	aa			
*F*_2_(NGB1238 × N24), markers linked to the *sym11* gene									
ZFP	30	47	0	0	1	30	0.0092	25.815	124.3951
Upl	32	43	2	0	5	26	0.0660	17.655	90.4198
RibL13l	31	46	0	0	1	30	0.0093	25.819	125.1111
AUXfp	33	43	1	0	4	27	0.0475	19.835	100.6667
PrOx	35	40	2	1	6	24	0.0970	14.582	80.3457
sdhFP	33	42	2	1	8	22	0.1159	12.656	66.1235
IPD3:c319del	30	47	0	0	0	31	0	28.284	133.3580
*F*_2_(NGB1238 × N24), markers linked to the *Sym13* gene									
PsC908p622	23	42	12	12	16	3	0.5	–	10.4198
PsC8268p528	26	39	12	9	19	2	0.5	–	11.4798
*F*_2_(SGE × N24), markers linked to the *sym11* gene									
RibL13l	7	11	0	0	0	9	0	7.667	42.6296
AUXfp	4	13	1	0	1	8	0.0640	4.723	31.2716
IPD3:c319del	7	11	0	0	0	9	0	7.667	42.6296

**Note:**

For each particular marker, “AA” means the number of plants carrying both alleles of a corresponding marker originated from the line NGB1238 or SGE; “Aa”—the number of heterozygous plants; “aa”—the number of plants carrying both alleles originated from the N24 line.

The localization of the *Sym11* gene in pea LG I is in contradiction with the previously reported location of the *Sym11* in pea LG VII (chromosome 7) by (Kneen, Weeden & LaRue, 1994). These authors based their map on the linkage of the *Sym11* gene with two isozyme markers Skdh (shikimic dehydrogenase) and Est-2 (esterase-2). The same pair of markers was also used by them for mapping of another pea symbiotic gene—*Sym13* (Kneen et al., 1990). Indeed, the *Sym13* gene was recently mapped relative to several gene-based molecular markers at LG VII (Kulaeva et al., 2017). To test whether both *Sym11* and *Sym13* genes might be in the same genomic region, we checked for co-segregation of the two markers of the LG VII region linked with the *Sym13* gene (PsC908p622 and PsC8268p528) and the *Sym11* gene. Analysis of the *F*_2_(NGB1238 × N24) mapping population clearly showed no linkage of both markers with the *Sym11* gene (see Table 3).

**Figure 3:**
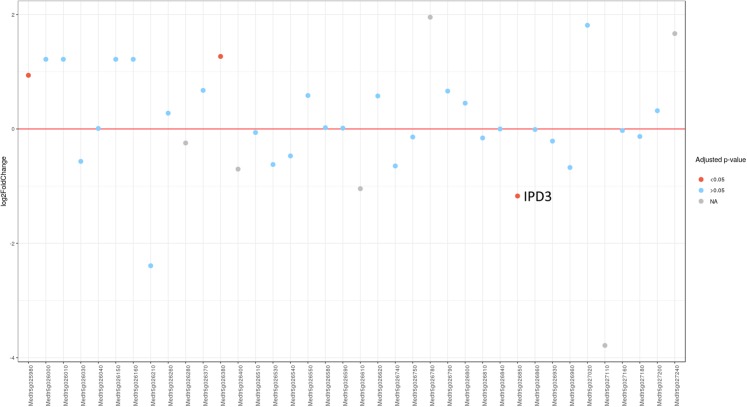
Differential expression of pea *Sym11* candidate genes. Pea *Sym11* candidate genes were chosen as homolog of Mt genes located in the defined genome region (see text). Each tick on the *x* axis represents a single pea transcript corresponding to Mt gene, which names are present on the *x* axis. *Y* axis represents the log2 fold change of gene expression in Fix^−^ in relation to Fix^+^ MACE libraries. Colors represent the adjusted *p*-value as calculated by the DESeq2 package (red < 0.05, blue > 0.05, gray—not calculated *p*-value). The only significant gene lying in the determined boundaries and with significantly lower expression in mutant nodules is pea homolog of *IPD3* (i.e., *Sym33*).

**Figure 4:**
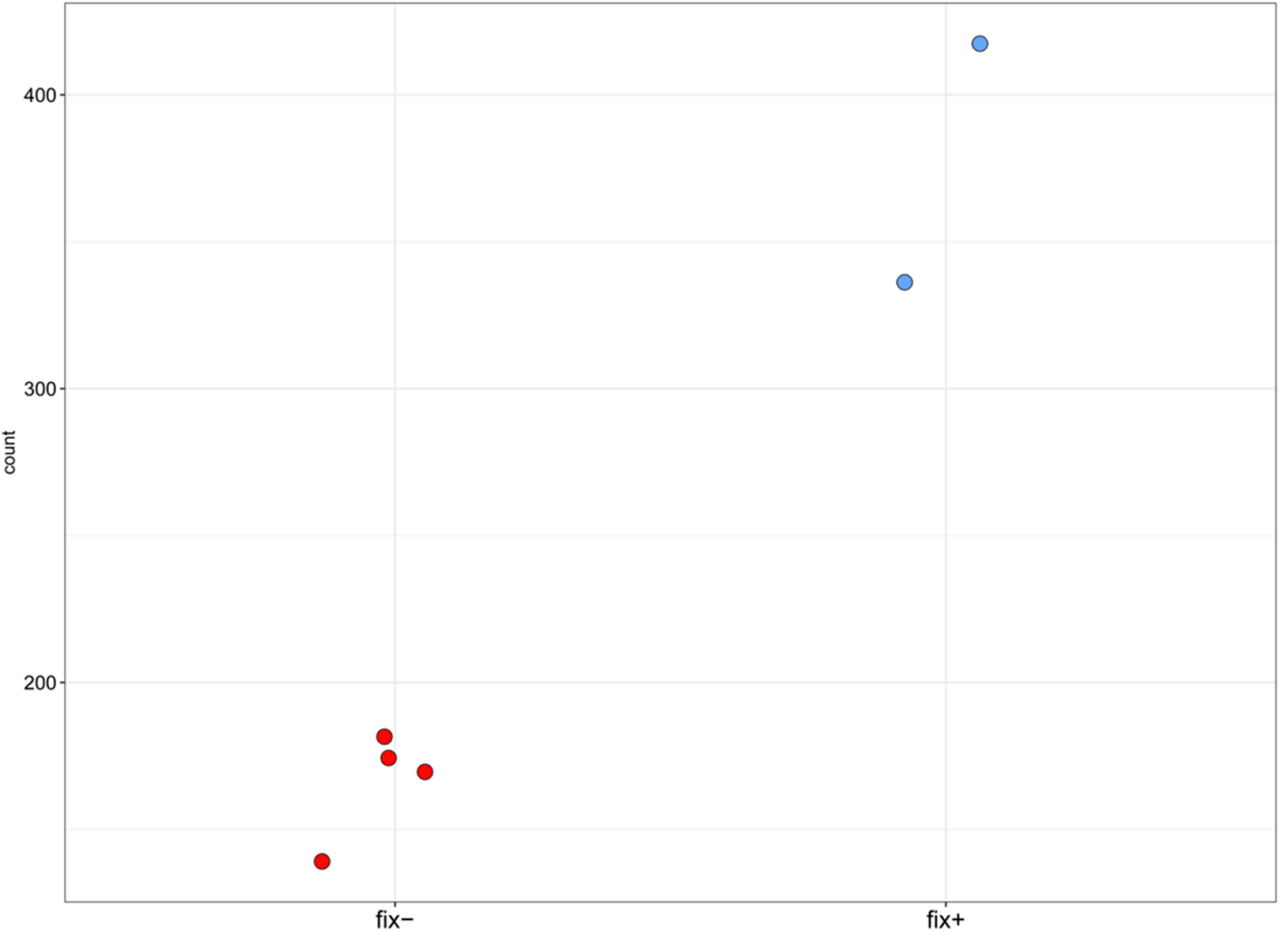
Normalized read counts for the transcript “GDTM01017250.1” (pea *Sym33*). *Y* axis shows the normalized read count for each sample. The blue markers represent the Fix^+^ samples, the red markers represent the Fix^−^ samples. *p*-value = 0.002948493.

Since MACE is a transcriptome profiling technology designed for differential gene expression analysis, we tried to explore its capacity for candidate gene search and analysis. To do this, we first defined the region in Mt genome containing genes homologous to markers ZFP and RibL13l and lengthened this interval by tripling the distance between these genes up and down the chromosome (see Table S3). Differential gene expression analysis between the phenotypically divergent bulks was performed. The PCA plot of normalized expression of samples produced by the DESeq2 package is represented on Fig. S3. The analysis revealed 2,235 significantly differentially expressed genes, of which 514 (23%) were up-regulated and 1,721 (77%) were down-regulated in plant with Fix^−^ phenotype. Then we determined the pea genes homologous to the Mt genes from the search region using BLASTN against PNTA with filtering the results by *e*-value (<1 × 10^−10^), query coverage (>70%), identity (>70%) and length (>300). Only three genes showed significant differential expression (adjusted *p*-value <0.05) (Fig. 3). Two up-regulated genes are homologous to Medtr5g025980.1, a putative bystin protein, and to Medtr5g026380.1, a hypothetical protein, both without known relation to symbiosis. The only down-regulated gene (Fig. 4) was homologous to the previously described *IPD3* gene of Mt (Messinese et al., 2007). The *IPD3* gene encodes a protein interacting with *DMI3*, which is a calcium- and calmodulin-dependent kinase essential for both rhizobial and mycorrhizal symbioses (Messinese et al., 2007). The *IPD3* gene is known in pea as *Sym33*, and *sym33* mutants form white nodules with no release of bacteria into the plant tissues, or develop nodule primordia only (the *sym33-1* and *sym33-2* mutant alleles (Ovchinnikova et al., 2011)), which is comparable with the phenotype variations characteristic for *F*_2_ homozygous *sym11* plants.

**Figure 5:**
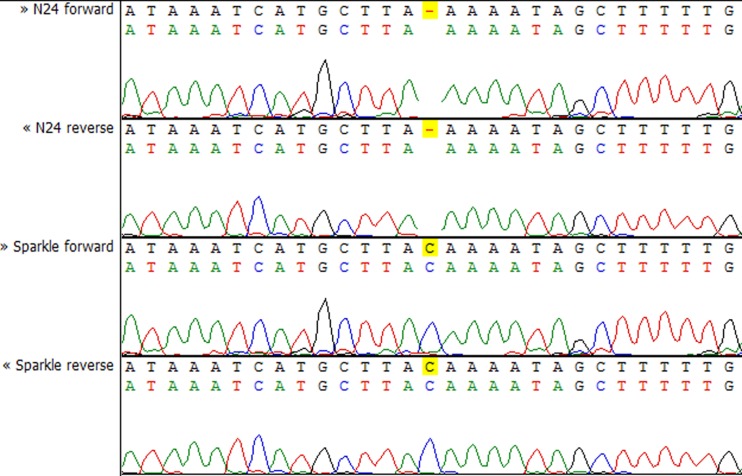
Sequencing chromatograms of the *IPD3* gene of lines N24 and Sparkle revealing the deletion harboring by N24.

To check whether the sequence of the *IPD3* gene contains a mutation in *sym11* mutant line N24, we sequenced *IPD3* of both the mutant and the corresponding WT parent cv. Sparkle. The exon-intron structure of the *IPD3* gene was constructed by aligning the sequenced contigs with the *IPD3* mRNA sequence (GenBank: EF569222.1) (see Fig. S2). Alignment revealed a mononucleotide deletion c.C319del in the third exon of the homologous sequence from the mutant N24 line (see Fig. 5 and Fig. S2), leading to a frame shift and premature translation termination after four erroneous amino acids (see Fig. S4). The putative truncated protein comprises 110 amino acids. The segregation analysis of the causal deletion C319del on both mapping populations showed its full co-segregation with mutant phenotype (see Table 3). This result implies that *Sym11* is, most probably, a new allele of *Sym33* (and may therefore be named *sym33-4*), although allelism tests with *sym33* mutants are necessary to fully confirm this suggestion.

## Discussion

In this study, we successfully mapped the *Sym11* gene of pea, using the combination of the MBS approach including bulk segregation analysis and MACE sequencing technology (MB-MACE). We performed the mapping on a rather small sample of 20 mutant-phenotype plants of the mapping population *F*_2_(NGB1238 × N24) distributed into four sequencing libraries. Thus, we identified a group of genes linked to the *Sym11* gene, that was confirmed by a segregation analysis of designed CAPS and dCAPS-markers on the extended mapping population *F*_2_(NGB1238 × N24) of 108 plants and another mapping population *F*_2_(SGE × N24) of 27 plants. The point RF estimates between the *Sym11* gene and the discovered genes putatively linked to it were calculated by two methods: by analysis of the ratio of polymorphic reads inherited from both parents in NGS-sequencing libraries and by analysis of segregation of markers in the mapping populations. The comparison of the two estimates showed that the first one is generally less reliable. The bias is caused, at least in part, by the errors in sequencing, demultiplexing and mapping of the NGS reads. Indeed, SNV-calling revealed NGB1238-originated reads in the sequencing libraries for the two genes closest to *Sym11*, ZFP (library M1, approx. 7%) and RibL13L (libraries M1, M2 and M4, approx. from 2% to 6%), (see Table S1, “Linkage estimation”). In reality (according to CAPS markers analysis), none of the M-libraries contained a plant with NGB1238 alleles of both markers. This erroneous occurrence of sequences from one library in the other is occasionally observed in NGS libraries due to the high number of sequences generated in parallel in the same NGS run and is called index hopping (Illumina, Inc., 2018). We here tried to prevent it by using two individual identifiers (barcodes) for each library. However, even this strategy obviously was not completely successful.

The “sliding window” approach is less sensitive to such errors but is applicable only if continuous genomic regions are available. The evaluation of genetic heterogeneity at a given site of a genome must be performed quite carefully. A lot of false-positive or overestimated linkages might be detected if suspect SNVs are not rejected. The information for rejection is received from the sequencing of control bulks, for which we can calculate the expected values of concentration of both alleles originated from each of parents. Significant deviation of observed polymorphic reads ratios from expected ones assuming complete linkage and the case of independent inheritance should be used for exclusion of SNVs (as it was done in our study).

Despite the difficulties described above, the MB-MACE approach can significantly reduce the cost of the MBS process compared to full-genome or full-transcriptome sequencing. In our case, sequencing of all six MACE libraries required only a quarter of a single Illumina lane. The proposed approach can be improved in the future, for example, by using normalization of cDNA libraries in order to decrease the number of abundant transcripts and increases the efficiency of random sequencing. On the other hand, non-normalized sequencing libraries may be immediately used for the candidate genes search by differential gene expression analysis between parent lines and the *F*_2_-plants.

Differential expression analysis did show a significant decrease in expression of the mutant gene in the roots of the Fix^−^ bulks. According to the *M. truncatula* Gene Expression Atlas (He et al., 2009) the *IPD3* gene is expressed in nodules and root hairs, so the downregulation of expression in these roots can be attributed to (1) the changes in the transcript that may lead to nonsense-mediated decay of the erroneous transcript and/or (2) the inability of the plants to form functioning nodules, which leads to changes in regulation for a large portion of symbiosis-related genes. Using MB-MACE, we unexpectedly, but unambiguously mapped the *Sym11* gene in the LG I, although in 1994 Kneen with colleagues (Kneen, Weeden & LaRue, 1994) positioned the *Sym11* gene at chromosome 7 (LG VII) basing on its linkage with Est-2 and Skdh isozyme markers. We suspect that the linkage of the *Sym11* gene with these two markers was false-positive due to the small size (totally 36 *F*_2_-offsprings) of the mapping population used by Kneen’s group, which also had skewed segregation toward the symbiotic phenotype (31 Nod^+^:5 Nod^−^: χ^2^-value = 2.37; *p*-value = 0.12), in addition to low number of markers used for mapping. Moreover, earlier mapping procedures were less precise, and a lot of condition-dependent markers, such as morphological or RFLP-based, were used. From time to time some markers were relocated in consensus linkage maps of the pea, including Est-2 and Skdh that were moved from chromosome 2 (LG II) to 7 (LG VII) (Weeden et al., 1998). The Skdh marker finally was linked to gene-based and SSR markers in linkage group VII (Loridon et al., 2005) and since that time had not been used. Interestingly, the Skdh marker intermediately helped mapping the *Sym13* gene of pea at the linkage group VII (Kulaeva et al., 2017).

Despite the closeness of the *Sym11* gene with two markers ZFP and RibL13l, approximately one cM away from each, the reliable arrangement of these loci cannot be established based on the available mapping populations (see Fig. 2). Nonetheless, such localization was useful for combined synteny and DGE based candidate search and candidate gene sequencing. Investigation of the corresponding Mt genome region (see Table S3, “Mt4 list”) revealed the candidate gene *IPD3*. The discovered c.C319 deletion (see Fig. 5) in the N24 allele of *IPD3*, which leads to an altered protein (see Fig. S4) and definitely co-segregates with the mutant phenotype (see Table 3), strongly supports our assumption.

Previously, three mutant alleles in the gene *sym33* were identified (Engvild, 1987; Tsyganov et al., 1998, 2013; Ovchinnikova et al., 2011). Coincidentally, the nucleotide deleted in N24 is exactly the same as the nucleotide changed in SGEFix^−^-5 (*sym33-2*) line (c.C319T) (Ovchinnikova et al., 2011). Nevertheless, the mutant SGEFix^−^-5 forms rare white small nodules (Tsyganov et al., 2013), whereas the mutant N24 displays no nodules. However, our observations showed that sometimes mutant SGEFix^−^-5 does not form any nodules (V. Tsyganov, 2016, personal observation). Evermore, Fix^−^ *F*_2_-plants from cross of N24 with NGB1238 line demonstrated the formation of nodules, and microscopic analysis revealed that infection threads were formed in the outer cortex cells but did not penetrate into the central zone of nodule, which remained empty. This phenotype is very similar to phenotype of nodules of the mutant SGEFix^−^-5 (Tsyganova, Ivanova & Tsyganov, 2019). Further studies are required to describe the manifestations of *sym33-4* allele in detail, including the investigation into the dependence of the phenotype on the rhizobial strain used for inoculation.

## Conclusion

The usage of the combination the MBS approach and MACE-Seq technology (MB-MACE) demonstrated its applicability for linkage mapping of pea genes in absence of a sequenced genome and using small bulk size (20 *F*_2_-plants). We particularly profited from the co-linearity—at least in the region of interest—of the pea and the Mt model genome, and from the fact that MACE-Seq identifies SNVs in genes rather than in anonymous sequences that enabled us to exploit this co-linearity. Even though the relatively small number of markers (217) had been checked for linkage with the pea (*P. sativum* L.) gene *Sym11*, that was enough to determine that the LG I region of the pea genome harbors the gene. At the current state in pea genetics, this approach is much less laborious, faster and cheaper than the traditional mapping techniques including individual marker screening. The combined synteny and DGE based analysis of the defined genomic region allowed us to identify the gene *IPD3* as a molecular essence of the *Sym11* gene. This result establishes the basis for further work on identification of the novel molecular functions of *IPD3* and its detailed role in symbiosis formation in pea.

## Supplemental Information

10.7717/peerj.6662/supp-1 Supplemental Information 1 UML Activity diagram outlining the major work steps.

10.7717/peerj.6662/supp-2 Supplemental Information 2 Alignment of partial genome sequences of gene IPD3 (lines N24 and Sparkle) and known mRNA of IPD3 (cv. Frisson, EF569222.1). The framed parts are sequences for annealing of primers used for amplification and sequencing. Red–forward, Blue–reverse.

10.7717/peerj.6662/supp-3 Supplemental Information 3 A PCA plot of normalized expression of samples produced by the DESeq2 package. Two blue rhombi represent fix+ samples, four red rhombi represent the fix- samples.

10.7717/peerj.6662/supp-4 Supplemental Information 4 Alignment of putative products of the IPD3 gene of lines Sparkle and N24.

10.7717/peerj.6662/supp-5 Supplemental Information 5 Genes for which the linkage analysis with the *sym11* gene was performed, including numbers of reads in each sequencing library, recombination frequency estimates and presumptive ortholog in *M. truncatula*. M1–M4–bulks composed from the Fix- and Nod- (recessive phenotype, N24 line) plants of the mapping population. W1, W2–bulks composed from the Nod+ (dominant phenotype, NGB1238 line) plants of the mapping population. The first number in the square brackets is the number of reads originated from the N24 pea line, the second–from the NGB1238 line.

10.7717/peerj.6662/supp-6 Supplemental Information 6 SNVs that were excluded from the linkage analysis due to the improbable observed ratio of reads in sequencing libraries. M1–M4–bulks composed from the Fix- and Nod- (recessive phenotype, N24 line) plants of the mapping population. W1, W2–bulks composed from the Nod+ (dominant phenotype, NGB1238 line) plants of the mapping population. The first number in the square brackets is the number of reads originated from the N24 pea line, the second–from the NGB1238 line.

10.7717/peerj.6662/supp-7 Supplemental Information 7 The list of *M.truncatula* genes located in the genomic region corresponding the genomic region of pea harboring the sym11 gene and their expression level. *M.truncatula* assemble version–Mt4.0v1, (Tang, et al. BMC genomics 15.1 (2014): 312.). The expression level for different organs and conditions is given according to https://phytozome.jgi.doe.gov/database.

## Additional Information and Declarations

### Data Availability

The following information was supplied regarding data availability: The raw data of NGSequencing is available in the SRA: PRJNA506113.
