# Supplementary figures and images for: Mapping-by-sequencing using NGS-based 3′-MACE-Seq reveals a new mutant allele of the essential nodulation gene Sym33 (IPD3) in pea (Pisum sativum L.)
Source: PeerJ. 2019 Apr 2;7:e6662. doi: 10.7717/peerj.6662 (PMC6450374; doi:10.7717/peerj.6662)

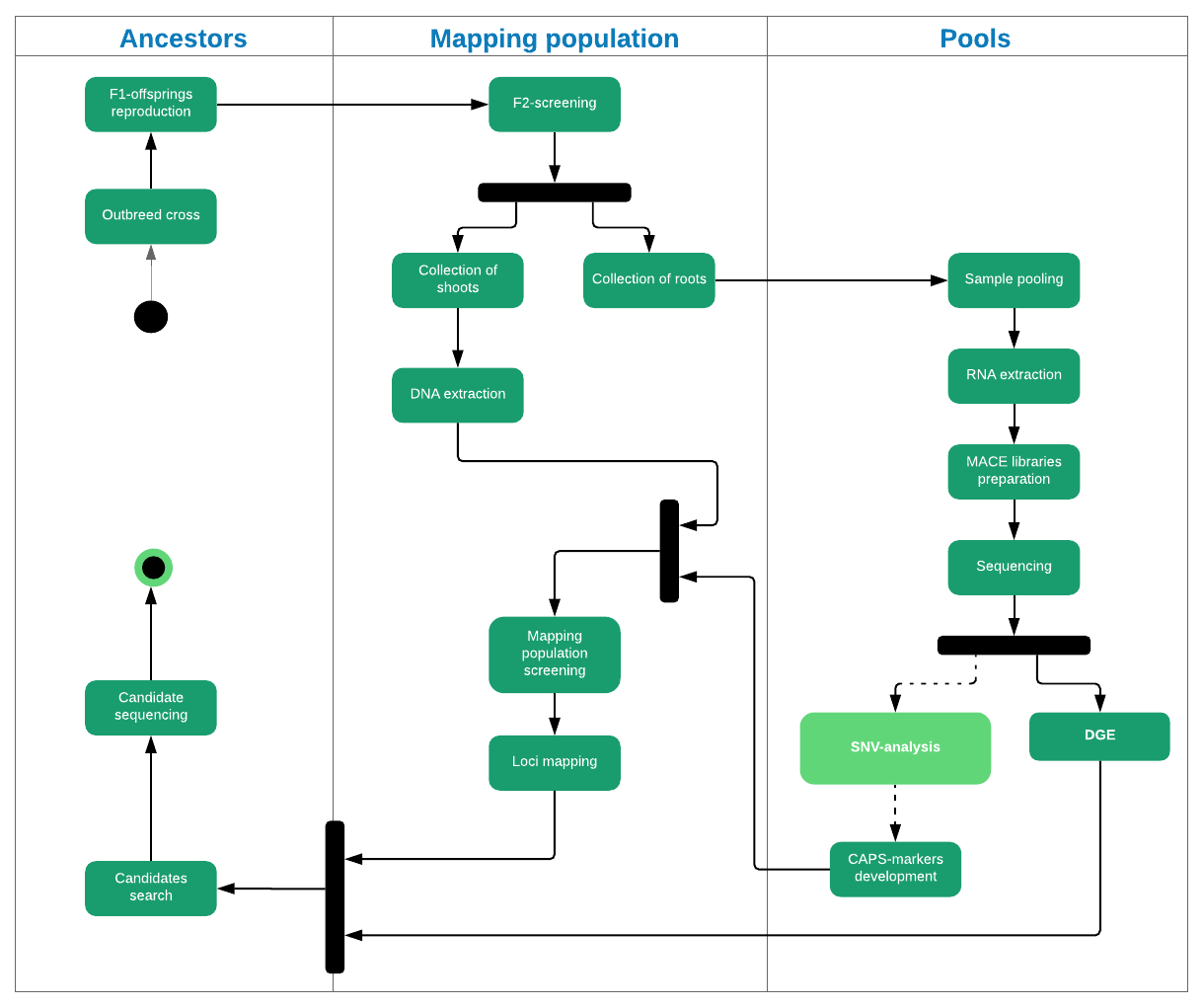

Supplement: Supplemental Information 1 [file peerj-07-6662-s001.png]

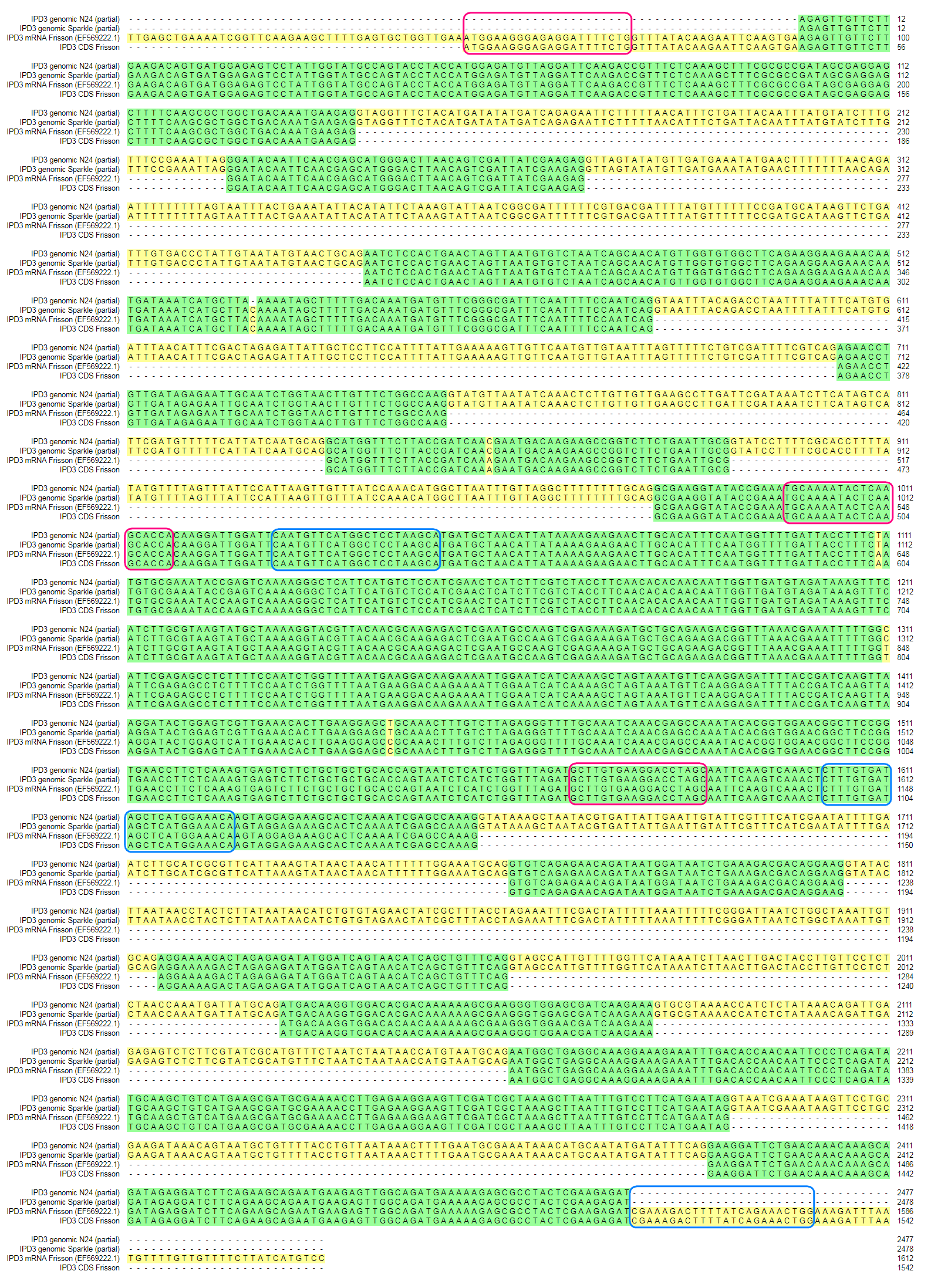

Supplement: Supplemental Information 2 — The framed parts are sequences for annealing of primers used for amplification and sequencing. Red–forward, Blue–reverse. [file peerj-07-6662-s002.png]

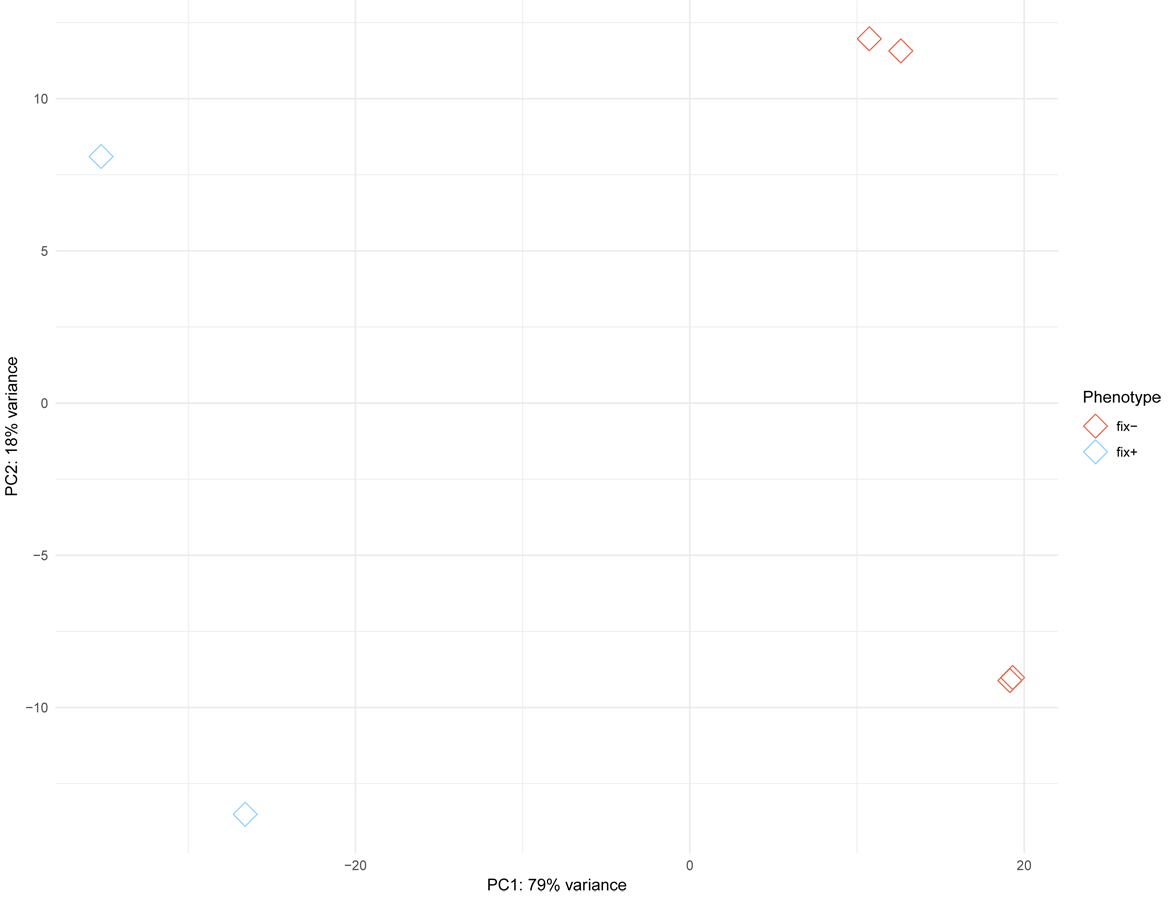

Supplement: Supplemental Information 3 — Two blue rhombi represent fix+ samples, four red rhombi represent the fix- samples. [file peerj-07-6662-s003.png]

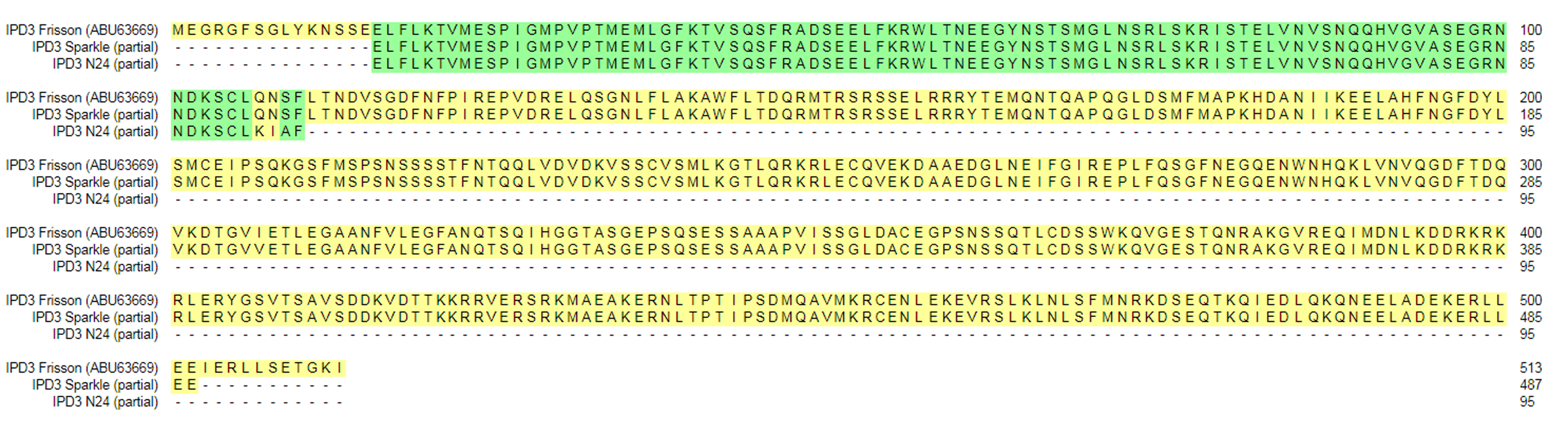

Supplement: Supplemental Information 4 [file peerj-07-6662-s004.png]
